# Avulsion Fracture of the Lesser Trochanter and the Use of Conservative Treatment

**DOI:** 10.7759/cureus.102252

**Published:** 2026-01-25

**Authors:** Dawid Bartosik, Bartlomiej Cwikla, Anna Kowalczyk, Michalina Loson-Kawalec, Anna Palka-Szymaniec, Bartosz Starzynski, Alina Keska, Jakub Szkuta, Klaudia Wojcik

**Affiliations:** 1 Faculty of Medicine, University of Opole, Opole, POL; 2 Department of Pediatric Surgery, University Clinical Hospital in Opole, Opole, POL

**Keywords:** avulsion fracture, conservative management, lesser trochanter, ossification center, rare injury

## Abstract

Avulsion fractures of the lesser trochanter of the femur are extremely rare injuries in the pediatric population, most commonly resulting from physical activity. The typical mechanism involves a sudden contraction of the iliopsoas muscle, which inserts on the lesser trochanter. We present a rare case of such an injury in a pediatric patient, emphasizing the importance of appropriate imaging and the effectiveness of conservative management in achieving full recovery.

An 11-year-old boy presented to the emergency department with perineal trauma sustained after losing balance while riding a bicycle and striking his perineum against the handlebars. He suffered a large laceration involving the pubic symphysis region, the base of the penis, and the right groin. Surgical wound exploration under general anesthesia revealed a 5-mm bone fragment near the damaged fascia of the quadriceps muscle. Postoperatively, the patient reported severe pain during attempts at right hip flexion, prompting further imaging. Pelvic radiographs and femoral CT scans revealed medial and superior displacement of the ossification center of the lesser trochanter of the right femur by 12 mm. Following multidisciplinary consultation with orthopedic surgeons, a conservative treatment approach was selected. The patient remained stable throughout treatment, showed progressive improvement, and achieved full functional recovery without the need for surgical intervention.

Avulsion fracture of the lesser trochanter is a rare but clinically significant injury in pediatric trauma care. This case highlights the importance of accurate imaging for diagnosis and demonstrates that conservative management can lead to complete recovery without surgical intervention.

## Introduction

An avulsion fracture of the lesser trochanter of the femur is an extremely rare injury in the pediatric population, accounting for less than 1% of hip region injuries [1]. A review of the literature, including the largest case series published by the Children's Hospital of Philadelphia, indicates that lesser trochanter fractures most commonly occur in children aged 9-17 years, predominantly in boys, and are primarily associated with physical activity [2]. The mechanism of injury is most often related to a sudden contraction of the iliopsoas muscle, which attaches to the lesser trochanter, leading to its avulsion [3,4]. We present a case of an 11-year-old boy diagnosed with an isolated avulsion fracture of the lesser trochanter of the femur.

## Case presentation

An 11-year-old boy presented to the emergency department with a perineal injury. The history revealed that the boy was riding a bicycle while standing on the pedals. After hitting an uneven surface, he lost his balance and struck his perineum against the handlebar. As a result, he sustained a large wound in the region of the pubic symphysis, the base of the penis, and the right groin. He was admitted to the pediatric surgery department of the University Clinical Hospital in Opole for further management on an emergency basis.

**Figure 1:**
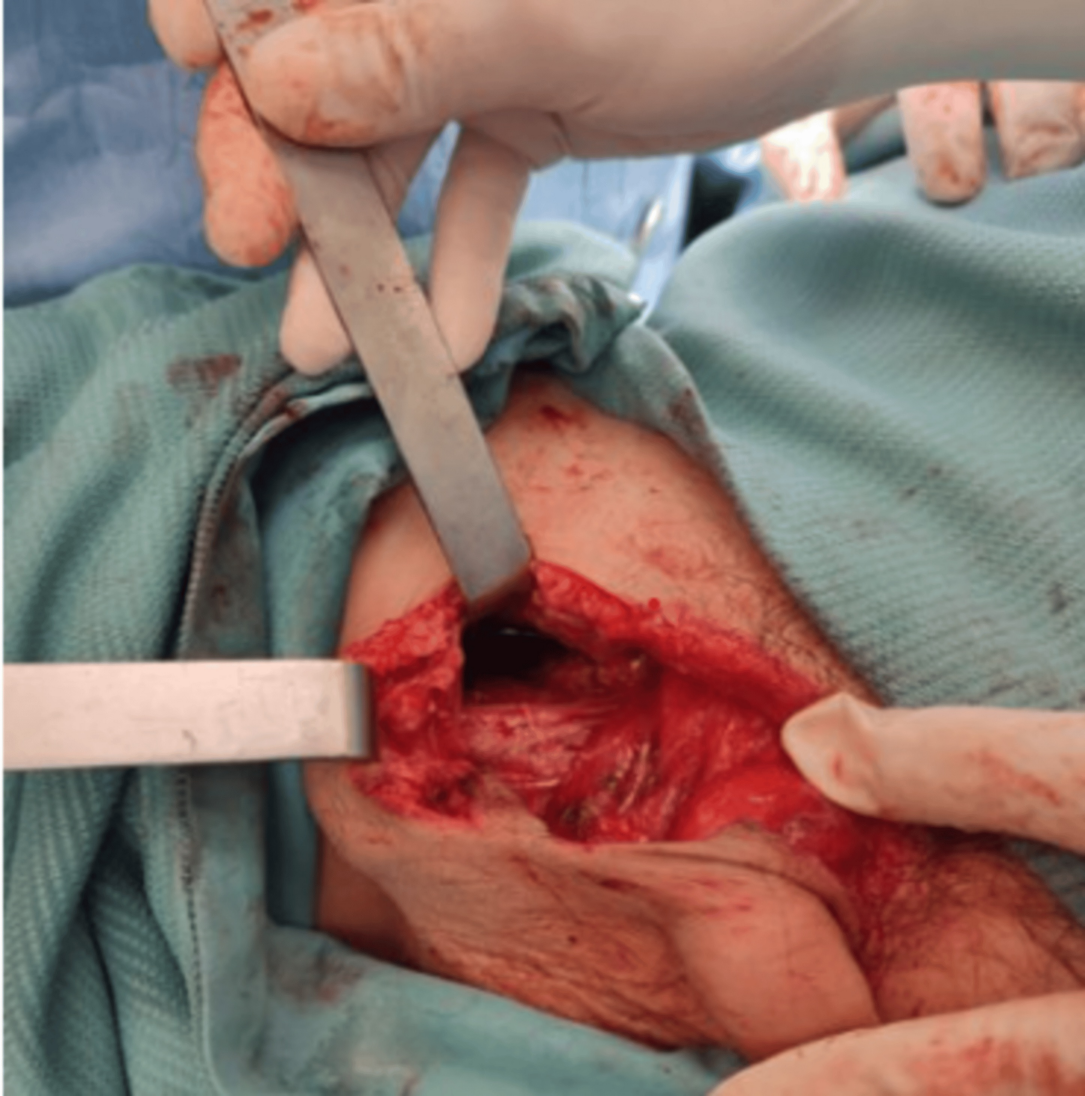
Intraoperative exploration

Initial treatment included antibiotic therapy and analgesia. The patient was then qualified for surgical intervention. Intraoperative exploration revealed skin damage and deep subcutaneous tissue injury extending toward the femur, as well as damage to the fascia of the quadriceps muscle (Figure 1). The spermatic cord and testis were exposed, but their coverings were intact. Additionally, a 5 mm bone fragment was identified near the damaged fascia of the quadriceps muscle. Palpation during the procedure did not reveal penetration of the wound into the bone. The bone fragment was removed.

**Figure 2:**
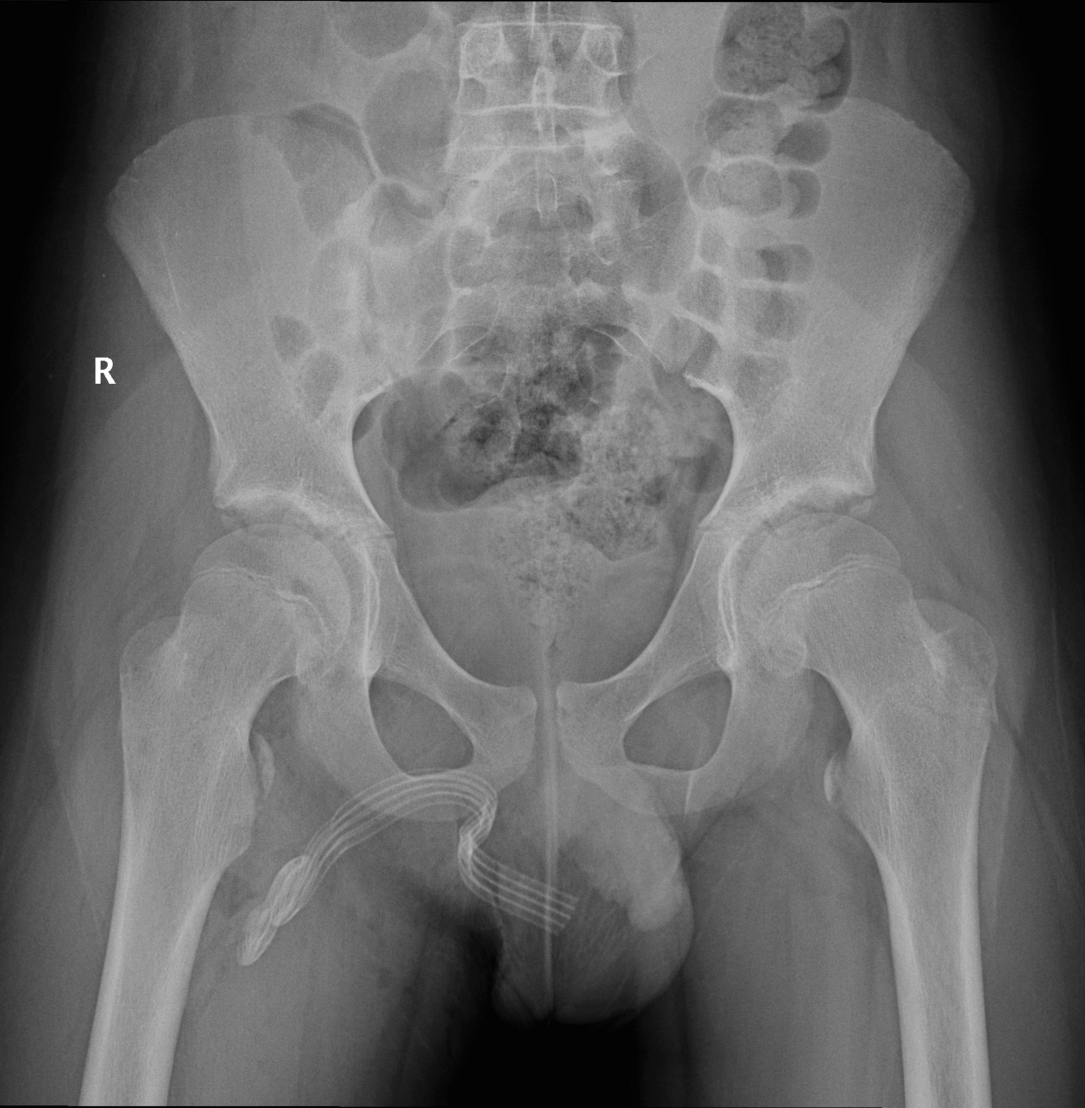
Pelvic X-ray after intraoperative exploration

**Figure 3:**
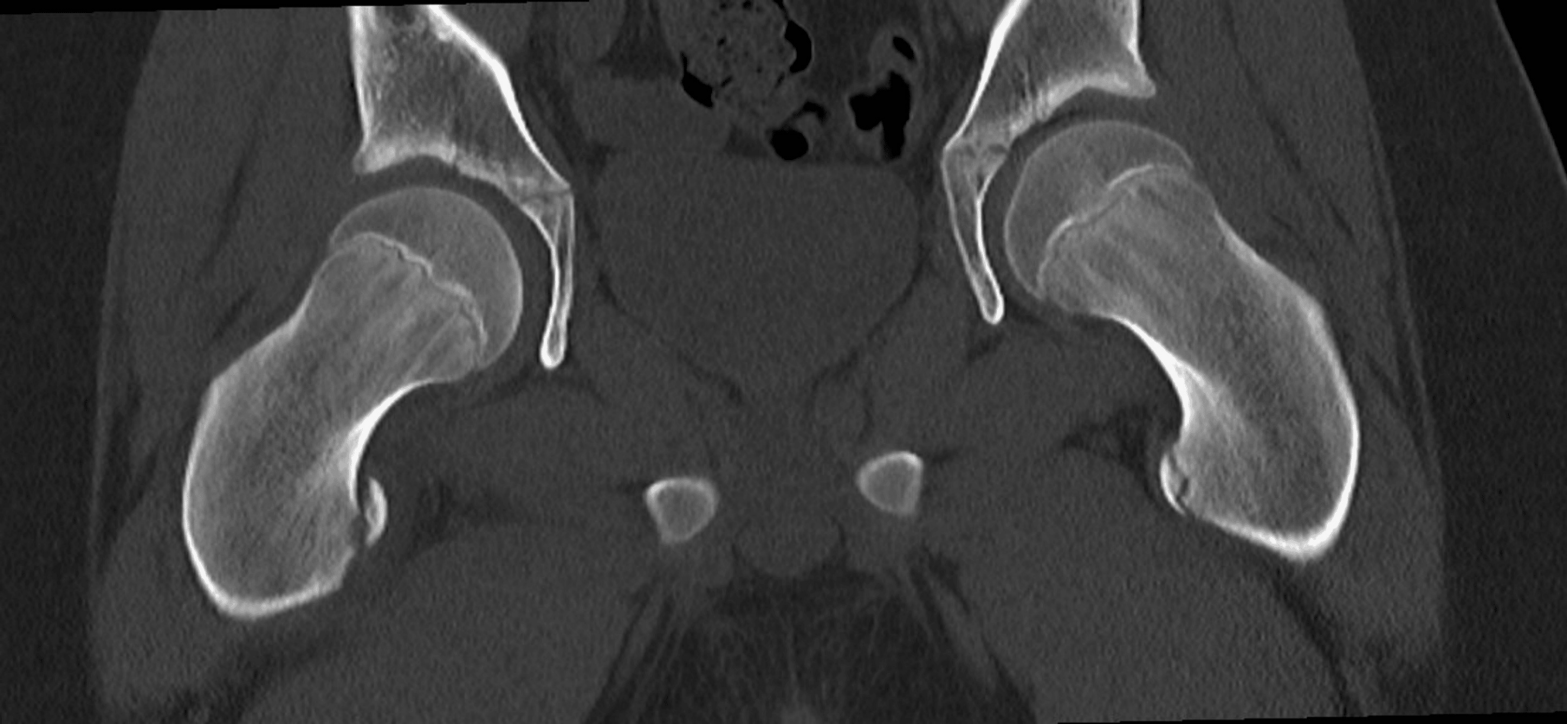
Pelvic CT after intraoperative exploration

Due to the findings and the patient’s severe pain during attempted flexion of the right hip joint postoperatively, imaging studies were performed. Pelvic X-ray (Figure 2) and femoral CT (Figure 3) revealed medial and superior displacement of the ossification center of the lesser trochanter of the right femur by 12 mm. In the Salter-Harris classification, this injury may correspond to type I, in which the displacement involves the cartilaginous epiphysis [3]. Conservative management of the fracture was chosen.

The wound healed well with no signs of infection throughout the healing period. Pain intensity gradually decreased. During hospitalization, the patient received instructions on proper crutch-assisted ambulation and appropriate analgesic therapy. Physical therapy of the hip joint and derotation of the limb in bed were also recommended.

**Figure 4:**
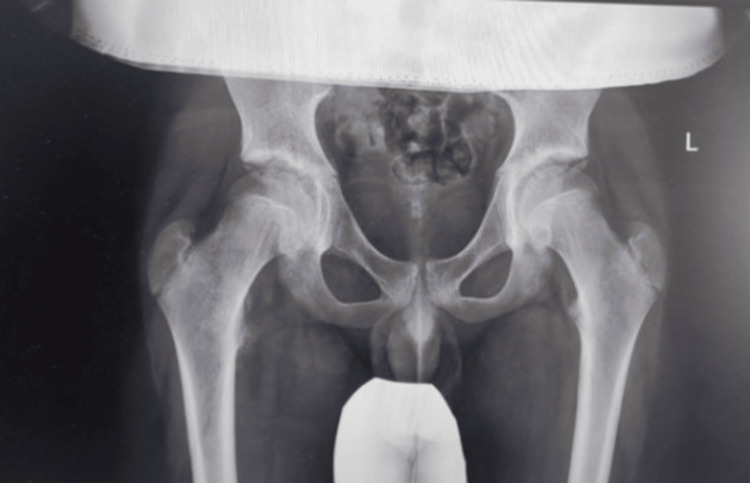
Pelvic X-ray three weeks after the injury

Three weeks after the injury, a follow-up X-ray performed in the outpatient clinic showed a stable fracture (Figure 4). Physiotherapy shortened the rehabilitation time, contributing to a faster recovery of the full range of motion in the right hip joint. Upon completion of therapy, the patient returned to full lower limb function without functional limitations or pain.

## Discussion

Avulsion fractures of the lesser trochanter are exceedingly rare. The ankle, distal femur, and distal radius are the most common sites of growth plate injuries [5]. A hospital in Philadelphia described 36 cases of children whose average age was 13.7 years; all of them sustained this type of injury during sports activities. These children were successfully treated with conservative treatment. Management consisted of limiting physical activity, unloading the limb, and rehabilitation with good clinical outcomes [2].

According to the Salter-Harris classification, there are five types of growth plate injuries. Most of them occur during periods of rapid growth, as growth plates are then most at risk [3]. Growth plates are cartilaginous structures that provide longitudinal bone growth during childhood and adolescence. Trauma or physiological or metabolic stress can lead to damage within these structures. The most frequent is injury during physical activity [5,6]. In adults, these types of fractures are rare and should prompt additional evaluation for possible metastatic bone disease [7]. In patients with this type of injury, a characteristic symptom is groin pain that worsens during hip flexion, resulting in a positive Ludloff test. An X-ray is usually sufficient to confirm the diagnosis and assess the degree of displacement. CT and MRI can show the injury in more detail, but they usually do not change the treatment plan. However, they may help detect other injuries caused by the trauma [8]. The choice between surgical and conservative management is a difficult clinical decision because of limited evidence and the rarity of these injuries [9]. Although conservative management is commonly used for these fractures, there are no clear indications regarding the optimal treatment. Published studies on this type of fracture mostly consist of small retrospective studies; some report excluding outcomes [10].

Various studies report that surgical treatment is indicated if fracture displacement exceeds 20 mm, symptomatic nonunion or exostosis develops, or if a patient cannot return to activities previous to injury [1]. Satisfying results have been reported with surgical treatment, whereas conservative treatment of lesser trochanter avulsions appears equally successful, with low rates of persistent symptoms or subsequent surgery [10].

Surgical management may provide a shorter initial recovery period. In the long term, results between conservative and surgical treatments may be similar. Therefore, the surgical method is recommended for people who want to quickly return to physical activity and require rapid rehabilitation, for example, athletes [11].

According to the literature, surgical treatment is indicated when the displacement exceeds 20 mm. This case supports the effectiveness of conservative treatment in patients with displacement up to 12 mm. It was confirmed on X-ray 30 days post-injury. Conservative treatment is less expensive and places a smaller burden on the patient, their family, and the healthcare system.

## Conclusions

Fractures of the lesser trochanter are uncommon in children but are clinically significant. Their rarity can make them difficult to recognize in pediatric trauma cases. This case emphasizes the importance of proper imaging diagnostics and conservative management to achieve full recovery. Effective treatment relies on proper immobilization, physiotherapy, and monitoring of the healing process through clinical and imaging follow-up. Further studies are needed to optimize management and rehabilitation strategies in such cases.
